# Antagonizing Bcl-2 Family Members Sensitizes Neuroblastoma and Ewing’s Sarcoma to an Inhibitor of Glutamine Metabolism

**DOI:** 10.1371/journal.pone.0116998

**Published:** 2015-01-23

**Authors:** Rachelle R. Olsen, Michelle N. Mary-Sinclair, Zhirong Yin, Kevin W. Freeman

**Affiliations:** Department of Oncology, St. Jude Children’s Research Hospital, Memphis, Tennessee, United States of America; Tulane University School of Medicine, UNITED STATES

## Abstract

Neuroblastomas (NBL) and Ewing’s sarcomas (EWS) together cause 18% of all pediatric cancer deaths. Though there is growing interest in targeting the dysregulated metabolism of cancer as a therapeutic strategy, this approach has not been fully examined in NBL and EWS. In this study, we first tested a panel of metabolic inhibitors and identified the glutamine antagonist 6-diazo-5-oxo-L-norleucine (DON) as the most potent chemotherapeutic across all NBL and EWS cell lines tested. Myc, a master regulator of metabolism, is commonly overexpressed in both of these pediatric malignancies and recent studies have established that Myc causes cancer cells to become “addicted” to glutamine. We found DON strongly inhibited tumor growth of multiple tumor lines in mouse xenograft models. *In vitro*, inhibition of caspases partially reversed the effects of DON in high Myc expressing cell lines, but not in low Myc expressing lines. We further showed that induction of apoptosis by DON in Myc-overexpressing cancers is via the pro-apoptotic factor Bax. To relieve inhibition of Bax, we tested DON in combination with the Bcl-2 family antagonist navitoclax (ABT-263). *In vitro*, this combination caused an increase in DON activity across the entire panel of cell lines tested, with synergistic effects in two of the N-Myc amplified neuroblastoma cell lines. Our study supports targeting glutamine metabolism to treat Myc overexpressing cancers, such as NBL and EWS, particularly in combination with Bcl-2 family antagonists.

## Introduction

Neuroblastoma (NBL), a pediatric embryonal malignancy of the developing sympathetic nervous system, and Ewing’s sarcoma (EWS), an aggressive malignancy of the bone and soft tissue, are devastating pediatric cancers. Only marginal improvements in outcome have been seen in both cancers over the past few decades, emphasizing the need for new therapies [1]. Targeting aberrant cancer metabolism including acquisition of aerobic glycolysis (Warburg effect), alteration of lipid synthesis, and addiction to glutamine offers a potentially exploitable “Achilles’ Heel” for treating NBL and EWS.

Oncogenesis and metabolism are intertwined with many core oncogenic pathways also controlling metabolism. Under most cellular conditions, signaling downstream of Myc, hypoxia-inducible factors (HIF) as well as the PI3K/Akt/mTOR pathway all favor cells utilizing glucose for various anabolic processes that permit rapidly proliferating cells to acquire an acute increase in biomass that facilitates cell division [2]. This helps explain the “Warburg Effect” where cancer cells import large amounts of glucose that is fermented into lactic acid even when oxygen is abundant (aerobic glycolysis)[3]. In addition to driving glycolysis, the PI3K/AKT/mTOR pathway and c-Myc turn the mitochondria into a factory for biosynthesis of lipids and nonessential amino acids derived from intermediates of the citric acid cycle (CAC) [2, 4]. However, this depletes the CAC of carbon, which is being inadequately resupplied by glycolysis due to the “Warburg Effect” [5]. To compensate for this depletion, carbon from glutamine can replenish the CAC via glutaminolysis, a two-step conversion of glutamine to glutamate and then from glutamate into the CAC intermediate alpha-ketoglutarate. While carbon from glutamine contributes to the CAC via glutaminolysis, glutamine is also broadly utilized in anabolic pathways to contribute nitrogen to the synthesis of nucleotides, amino acids, and hexosamines [6].

c-Myc causes glutamine addiction in multiple cell lines, and recently N-Myc has been shown to have a similar effect in NBL [7, 8]. Amplification of *MYCN*, a c-Myc family member, is associated with both poor prognosis and high-risk disease in NBL [9]. EWS is caused by chromosomal translocations that result in expression of EWSR1/ETS fusion proteins. The most common fusion found in 85% of EWS is the EWSR1/FLI fusion that targets c-Myc for overexpression and may cooperate with c-Myc in transforming cells [10, 11]. Myc has been shown to coordinate glutamine metabolism by regulating a number of genes that import and utilize glutamine including the glutamine transporters ASCT2 and LAT1, glutaminase 1, and multiple nucleotide biosynthetic genes [12, 13]. Though Myc has proven intractable to direct drug targeting, targeting the consequences of Myc transformation, such as an altered cellular metabolism, may hold promise [14, 15].

In this study we tested a panel of metabolic inhibitors that target four core metabolic pathways which are altered in cancer: glycolysis, glutamine metabolism, fatty acid metabolism, and lactic acid production [4, 6, 16, 17]. From this screen the most effective inhibitor we identified was 6-diazo-5-oxo-L-norleucine (DON), a well-characterized small molecule that irreversibly inactivates glutamine-utilizing enzymes and is a potent inhibitor of glutamine metabolism. DON was first explored as a cancer chemotherapeutic in the 1950s and 1960s and was found to cause occasional clinical responses, but also nausea [18]. However, by medicating with the antiemetic chlorpromazine, a phase I pediatric study from 1983 achieved therapeutic levels of DON without the associated nausea [19]. In the pediatric study, all six solid tumor patients that received a >300 mg/m^2^ twice-weekly dose of DON showed improvements. Since the relationship between Myc, glutaminolysis and glutamine addiction was unknown at that time, DON’s main mechanism of action was attributed to inhibition of nucleotide synthesis.

In this study, we identified glutamine metabolism as a vulnerability of both NBL and EWS. We further performed an assessment of DON against NBL and EWS tumors to determine whether targeting glutamine metabolism is a viable therapeutic approach that should be pursued for neuroblastoma (NBL) and Ewing’s sarcoma (EWS). Our results show that high-Myc expressing tumors are susceptible to DON-induced apoptosis and suggest that combining inhibitors to glutamine metabolism with antagonists to Bcl-2 family members could be a successful treatment strategy for these pediatric cancers.

## Materials and Methods

### Cell Culture

BJ fibroblasts were purchased from ATCC, and were maintained in EMEM media supplemented with 10% FBS, 1% Penicillin/Streptomycin, 2mM L-glutamine, 1mM sodium pyruvate, and 1.5g/L sodium bicarbonate. Kelly cells were purchased from Sigma-Aldrich, and were maintained in RPMI-1640 media supplemented with 10% FBS, 1% Penicillin/Streptomycin, and 2mM L-glutamine. All remaining cell lines were obtained from ATCC and maintained as described below: IMR32 and SK-N-MC cells were cultured in EMEM supplemented with 10% FBS, 1% Penicillin/Streptomycin, and 2mM L-glutamine. SK-N-FI and SK-N-AS cells were cultured in DMEM (1g/L glucose) supplemented with 10% FBS, 1% Penicillin/Streptomycin, 1.5g/L sodium bicarbonate, and 4mM L-glutamine. SK-SY5Y and SK-N-BE2 cells were cultured in 1:1 EMEM/Ham’s F-12 media supplemented with 10% FBS, 1% Penicillin/Streptomycin, and 2mM L-glutamine. RD-ES cells were cultured in RPMI-1640 supplemented with 15% FBS, 1% Penicillin/Streptomycin, and 2mM L-glutamine. SK-ES-1 cells were cultured in McCoy’s 5A media supplemented with 10% FBS, 1% Penicillin/Streptomycin, and 2mM L-Glutamine. All cell lines were grown at 37°C in a humidified atmosphere with 5% CO_2_. Identity of all cell lines was verified by both STR analysis and karyotyping at the St. Jude Hartwell Center for Bioinformatics and Biotechnology and the St. Jude Cytogenetics Lab, respectively. Additionally, all cell lines were PCR tested and shown to be mycoplasma free.

### CyQuant Assay

The following metabolic inhibitors were obtained from Sigma-Aldrich and solubilized in water: 6-diazo-5-oxo-L-norleucine (DON, #D2141), Oxamate (#O2751), Bromopyruvate (#16490) Dichloroacetate (#347795), Etomoxir (#E1905), and Trimetazidine (#653322). Bezafibrate (Sigma-Aldrich, #B7273) was dissolved in methanol. Lonidamine (Sigma-Aldrich, #L4900) and ABT-263 (Selleckchem) were dissolved in 100% dimethyl sulfoxide (DMSO). In a 96-well tissue culture plate, 5x10^4^ cells were plated in 100µl media containing EMEM, 10% FBS, and 0.5mM L-glutamine per well. Four hours after plating, drugs were diluted to a 2x concentration in plating media and then 100µL was added to each well. Plates were incubated for 72 hours and then submitted to CyQuant Cell Direct Proliferation Assay (Life Technologies) according to manufacturer’s instructions and read with a Synergy HT Multi-mode microplate reader (Bio-Tek).

### Western Blotting and Antibodies

MPER (Mammalian Protein Extraction Reagent, Pierce ThermoScientific) was used to make protein lysates from sub-confluent cultures of all cell lines using manufacturer’s protocol. Lysis buffer was supplemented with Halt Protease and Phosphatase Inhibitor Cocktail (Pierce/ThermoScientific) and protein concentrations were measured with the BCA Assay (Pierce/ThermoScientific). Lysate volumes containing equal protein were loaded on polyacrylamide gels (Bio-Rad) then transferred to 0.2 µm PVDF membranes (Invitrogen) using a Semi-Dry Transfer system (Bio-Rad). Membranes were blocked in 5% milk/PBST for one hour, followed by primary antibody incubation overnight at 4°C. Antibodies used include the following: cMyc (1:200, R&D #AF3696), NMyc (1:1000, Cell Signaling Technology #9405), β-tubulin (1:1000, Cell Signaling Technology #2146), Bax (1:8000, Cell Signaling Technology #2772), Bak (1:2000, Cell Signaling Technology #3814), Actin (1:50000, Sigma-Aldrich, clone AC-15 #A5441), anti-goat-HRP (1:2000), anti-rabbit-HRP (1:2000, Cell Signaling Technology #7074), and anti-mouse-HRP (1:10000, Cell Signaling Technology #7076). Membranes were developed using ECL Plus or ECL Prime (GE Healthcare/Amersham) and exposed to HyBlot CL autoradiography film.

### Animal Experiments

This study was performed in strict accordance with the recommendations in the Guide for the Care and Use of Laboratory Animals of the National Institute of Health. All animal experiments were approved by the Institutional Animal Care and Use Committee (IACUC) at St. Jude Children’s Research Hospital. For subcutaneous tumor experiments, cells were injected into the hind flank of 6–8 week old athymic (nu/nu) mice (Charles River). SK-N-AS, SK-N-MC, SK-ES-1, and SK-N-FI cells were injected at 2x10^6^ cells per mouse in 100µl total volume. SK-N-BE2 and IMR32 cells were injected in a 1:1 mix of cells and Matrigel (BD Biosciences) at a final concentration of 2x10^6^ cells per mouse. Tumor size was measured by digital caliper, and tumor volume was calculated using the formula 1/2 * (length * width^2^). When tumors reached an average size of 200 mm^3^ mice were randomized into treatment groups. Five mice per treatment group for SK-N-AS; three mice per treatment group for SK-N-FI, SK-N-MC, and SK-ES-1; ten mice per treatment group for SK-N-BE2 and IMR32. On the day of treatment, mice were weighed then given an intraperitoneal (i.p.) injection of 100 mg/kg DON, 50 mg/kg DON or water control. Mice were housed with 4 to 5 mice per cage with corn cob bedding in normal light-dark cycling at room temperature (~23°C). Food and water were provided ad libitum. Mice were sacrificed using CO_2_ asphyxiation followed by cervical dislocation.

### BrdU Tumor Experiments

For *in vivo* labeling of proliferating cells, mice with subcutaneous SK-N-AS or SK-N-BE2 tumors were treated with DON and injected with BrdU (BD Biosciences). Mice were given an intraperitoneal (i.p.) injection of DON and 4 hours later mice were injected i.p. with 150µL of 10mg/ml BrdU. Tumor tissue was harvested 2 hours after addition of BrdU and samples were fixed in 10% formalin. Paraffin-embedded tissue sections were then stained for BrdU incorporation and cleaved caspase 3 by the St. Jude Veterinary Pathology Core. For each tumor ten images were captured, quantified by NIS-Elements BR imaging software and then averaged for both BrdU and caspase 3 positive staining. Statistical significance was determined using t-test in Excel 2010.

### NMyc Overexpression

Non-tissue culture treated 96-well plates were coated with 25µg/ml retronectin (Takara #T100B) in PBS for 2 hrs at room temperature, followed by incubation for 45min in 2% BSA/PBS. After washing with PBS, 200µL of pLOVE-NMyc (Addgene plasmid #15951) lentivirus was added per well, and then the plate was spun down at 1000G for 60min at room temperature. Wells were washed with PBS, and then 2.5x10^5^ SK-N-AS cells were plated per well in 200µL total volume. Cells were expanded as needed to create stable pools, and no selection reagents were used. For CyQuant assay, 1.7x10^4^ cells were plated in 96-well plates in 100µL EMEM media containing 10% FBS, 0.5mM glutamine, and 1% penicillin/streptomycin. Four hours later, 100µL of 2x DON was added. Cells were analyzed by CyQuant assay as described above following 72hr DON treatment. Statistical analysis was performed by ANOVA (GraphPad).

### QVD Caspase Inhibitor Experiments

Pan-caspase inhibitor QVD was purchased from Sigma-Aldrich (SML0063) and solubilized in DMSO. Neuroblastoma cells were plated in 96-well plates at 5x10^4^ cells/well (FI, AS, SY5Y, BE2 and IMR32) or 3x10^4^ cells/well (Kelly) in 100µl media containing EMEM, 10% dialyzed FBS, 0.5mM L-glutamine, and 1% Penicillin/Streptomycin. Four hours later, cells were treated with 100µl of 2x DON media +/- 2x QVD (final concentration 20µM). We compared simultaneous addition of DON and QVD for 72hr hours to sequential addition of QVD after 24hr DON treatment (48hr total QVD). Following 72hr DON treatment, cells were analyzed by CyQuant assay as described above. Statistical analysis was performed by ANOVA (GraphPad).

### Knockdown of Bax and Bak

shRNAmiR plasmid sets for knockdown of human Bax (TRHS1000–581) and Bak (TRHS1000–578) were purchased from TransOmic, along with Non-Targeting shRNA-miR negative control. For production of VSV-G pseudotyped retrovirus, 293T cells were co-transfected with shRNA-miR plasmid, VSV-G envelope, and Gag/Pol using Trans-IT293 (Mirus Bio). Virus-containing media was harvested 48hr post-transfection and filtered through a 0.45µm polyethersulfone (PES) filter. Viral titer was measured by FACS analysis in 293T cells infected with 1:1,000 or 1:10,000 dilutions of virus to detect expression of IRES-TurboGFP from the vector backbone. For knockdown of Bax and Bak, 5x10^5^ Kelly cells were infected with 1x10^6^ viral particles (MOI = 2). One week following infection, cells were plated for CyQuant assay and remaining cells were lysed for western blot analysis. For CyQuant, cells were plated in 96-well plates at 3x10^4^ cells/well in 100µL EMEM, 10% dialyzed FBS, 0.5mM L-glutamine, 1% Penicillin/Streptomycin media, followed by treatment 4 hours later with 100µL of 2x DON media. CyQuant assay was performed after 72hr DON treatment, as described above. Statistical significance was determined by two-way ANOVA (GraphPad).

## Results

### 6-Diazo-5-oxo-L-norleucine (DON) is an effective metabolic inhibitor in NBL and EWS

**Figure 1:**
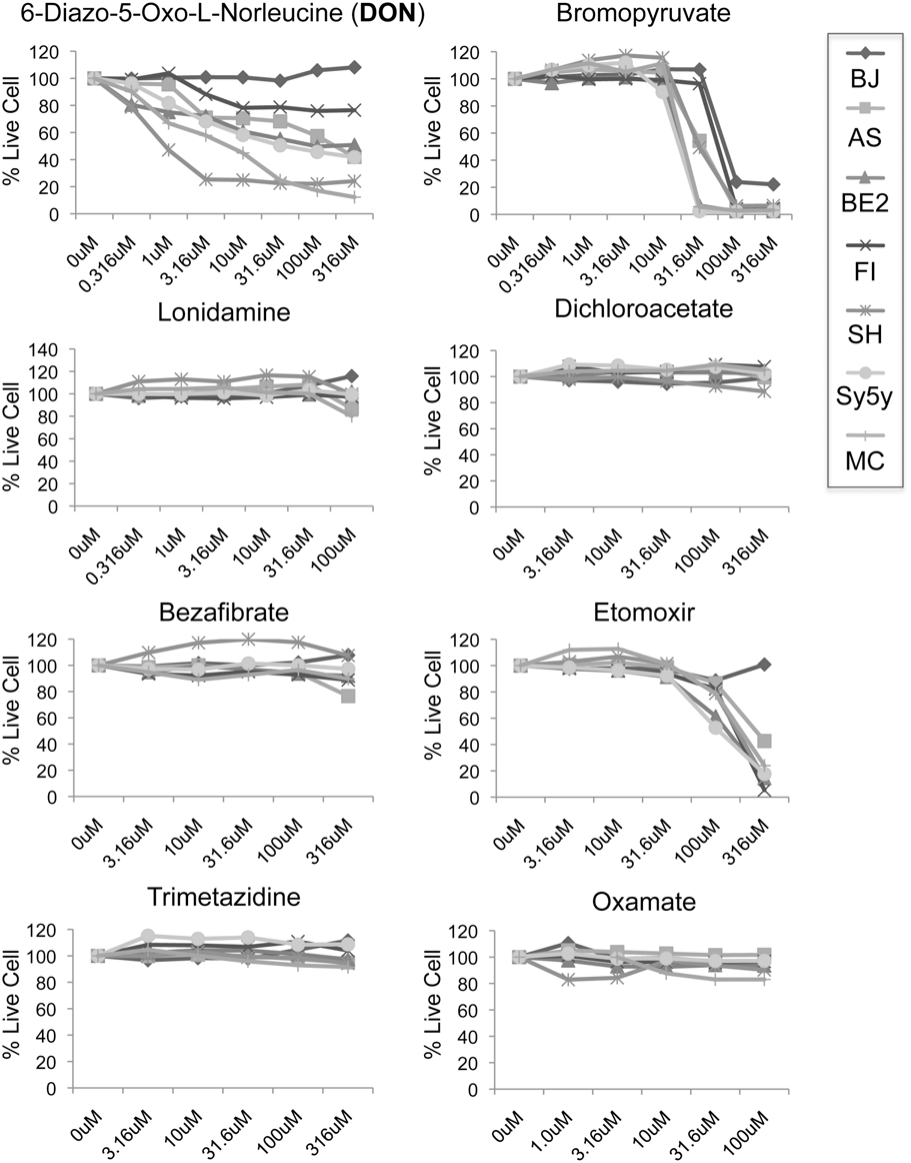
The glutamine antagonist 6-diazo-5-oxo-L-norleucine is the most effective metabolic inhibitor tested against NBL and Ewing’s sarcoma cell lines. Cells were treated with a dose response curve of the indicated inhibitor for 72 hrs prior to measuring cell viability by CyQuant assay. Control BJ cells were used to test for overt toxicity. Data is shown as percent of control (% Live Cells), and is representative of three independent experiments.

To probe the metabolic susceptibilities of NBL we screened a panel of NBL cell lines with multiple metabolic inhibitors. We prioritized inhibitors that have established *in vivo* activity to permit later *in vivo* testing. The metabolic pathways we targeted include **glycolysis** (bromopyruvic acid, lonidamine, and sodium dichloroacetate), **glutamine metabolism** (6-diazo-5-oxo-L-norleucine (DON)), **fatty acid metabolism** (bezafibrate, etomoxir, trimetazidine) and **lactic acid production** (oxamate) [4, 16, 17]. Five neuroblastoma cell lines (SK-N-AS, SK-N-BE2, SK-N-FI, SK-N-SH and SY5Y), one Ewing’s sarcoma cell line misidentified by ATCC as a neuroblastoma cell line (SK-N-MC) [20] and a control immortalized foreskin fibroblast cell line (BJ), which does not exhibit a Warburg effect [21], were used in the initial screen. Using the CyQuant viability assay we determined the respective sensitivities of each cell line to agent after 72 hr treatment with inhibitors in a universal media (EMEM, 10% FBS, 0.5mM Glutamine) containing physiological levels of glutamine and glucose (Fig. 1). Both the glycolysis inhibitor bromopyruvate and the carnitine palmitoyltransferase-1 inhibitor etomoxir reduced percent live cell count but only at the highest concentrations. Bromopyruvate also caused toxicity in BJ fibroblasts suggesting it is generally toxic at those levels. More promising results were seen with DON, which caused as much as a 90% reduction in percent live cell count, while leaving BJ cells unaffected.

The ability of DON to inhibit cell viability was then confirmed using a broader panel of six neuroblastoma cell lines (SK-N-AS, SK-N-BE2, SK-N-FI, IMR32, Kelly, and SY5Y) (S1A Fig.), three Ewing’s sarcoma cell lines (SK-N-MC, RD-ES and SK-ES-1) (S1B Fig.), and BJ cells. With the exception of the control BJ cells, all cell lines showed some sensitivity to DON. SK-N-FI cells were the most resistant with only a 20% reduction in cell number while multiple cell lines showed greater than 60% loss in cell number (S1A Fig.). All three Ewing’s sarcoma cell lines were especially sensitive to DON with an 80% loss in cell viability (S1B Fig.).

### DON inhibits tumor growth

**Figure 2:**
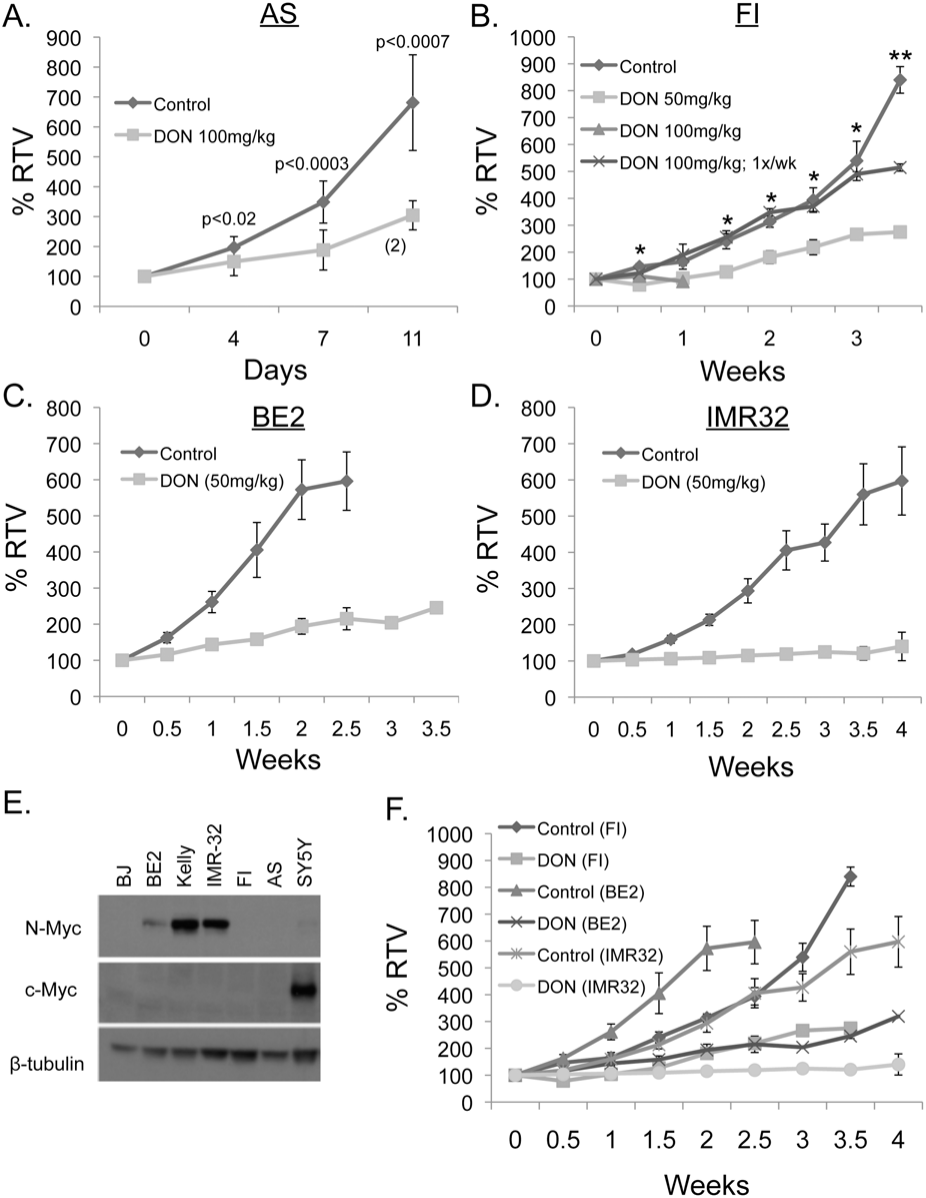
DON significantly inhibits xenograft tumor growth. (A) SK-N-AS tumors were treated with DON at 100 mg/kg or water by i.p. twice weekly. Weight loss in DON-treated mice reduced the treatment cohort to 2 mice indicated by (2) at later timepoints. (B) SK-N-FI tumors were treated with DON at 100 mg/kg, 50 mg/kg or water by i.p. twice weekly or 100mg/kg once a week (100mg/kg; 1x/wk). The * indicates p< 0.05 between control and 50 mg/kg, while ** indicates p<0.05 between control and 100mg/kg; 1x/wk, Student’s *t* test. (C and D) For SK-N-BE2 and IMR32 subcutaneous xenografts, mice were treated with DON at 50 mg/kg or water control twice weekly by i.p. injections. The indicated tumor cell line was grown to 200 mm^3^ prior to initiation of treatment and tumor size is given as a percent relative to original tumor volume (% RTV). (E) Western blot analysis of N-Myc and c-Myc expression in a panel of NBL cell lines with β-tubulin as a loading control. (F) A composite graph of SK-N-FI, SK-N-BE2 and IMR32 tumors treated with DON 50mg/kg or water control twice weekly.

To assess the effects of DON on tumor growth we treated subcutaneous tumors for the neuroblastoma cell line SK-N-AS as well as the Ewing’s sarcoma cell lines SK-N-MC and SK-ES-1. Tumors of ~200 mm^3^ in size were then randomized into either DON or control treatment groups. Mice were treated twice a week by intraperitoneal (i.p.) injection with 100 mg/kg DON (the mouse equivalent to the 300 mg/m^2^ dose previously used in children) or water control. In the DON pediatric clinical study a dose as high as 520 mg/m^2^ twice a week was safely achievable in children [19]. At the 100mg/kg dose, DON significantly reduced growth of both neuroblastoma SK-N-AS tumors (Fig. 2A) and the EWS tumors SK-N-MC and SK-ES-1 (S2A and S2B Figs.). However, DON at 100mg/kg caused weight loss in the mice that limited the extent of treatment.

To ameliorate the weight loss seen in mice, we compared the effectiveness of the original drug regimen to two new treatment schedules. We used subcutaneous tumors from the most DON-resistant cell line, SK-N-FI, reasoning that any treatment schedule that showed efficacy against SK-N-FI would likely show efficacy against the other cell lines. To attempt to extend the course of treatment, mice were either treated with half the dose at 50 mg/kg with the same schedule of twice a week, or with a once weekly injection of 100 mg/kg (1x/wk). Both of the new drug regimens allowed a significant extension in length of treatment from 11 days to greater than 28 days (Fig. 2B). However, only the 50 mg/kg twice a week dose slowed tumor growth (Fig. 2B). Interestingly, DON was more effective against SK-N-FI tumors than might be expected based on our *in vitro* results.

To assess additional NBL tumors, we then tested the 50mg/kg twice weekly dose on two N-Myc amplified NBL cell lines (SK-N-BE2 and IMR32). We saw significant differences in tumor growth rates when mice were treated with DON as seen for tumor averages + SE shown (Fig. 2 C, D). On average SK-N-BE2 tumors treated with DON were 3-fold smaller at 2.5 weeks of treatment (Fig. 2C). For IMR32 tumors, DON on average completely inhibited tumor growth leading to tumors that were 6-fold smaller than control tumors (Fig. 2D). In all tumor studies, DON treatment significantly reduced growth of tumors, including SK-N-FI tumors, suggesting that inhibitors of glutamine metabolism could be broadly efficacious against NBL and EWS.

Since Myc is known to cause glutamine addiction, we next assessed whether Myc expression levels could be responsible for the greater DON sensitivity of IMR-32 tumors versus BE2 and FI tumors. Of these three cell lines, western blot analysis showed that IMR32 cells express the highest levels of Myc (Fig. 2E). A composite graph of the tumor studies for SK-N-FI (Fig. 2B), SK-N-BE2 (Fig. 2C) and IMR32 (Fig. 2D) is shown in Fig. 2F and confirms that the IMR32 cell line was the most sensitive to DON *in vivo*. BE2 tumors with moderate N-Myc levels (Fig. 2C and 2E) and SK-N-FI tumors with no observable N-Myc expression (Fig. 2B and 2E) showed comparable sensitivity at this dose of DON.

### DON is generally cytostatic, but is specifically pro-apoptotic in high N-Myc tumors

**Figure 3:**
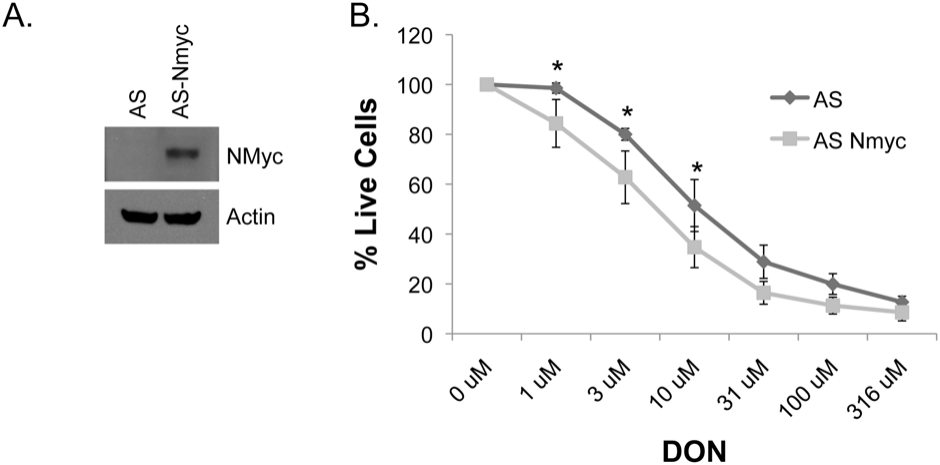
NMyc overexpression sensitizes cells to DON. (A) SK-N-AS cells were infected with pLOVE-NMyc lentivirus, and then NMyc overexpression was determined by western blot. (B) Dose response curve comparing 72hr DON treatment in SK-N-AS cells with varying levels of NMyc. Data is shown as average ± stdev from three independent experiments. Statistical significance was determined by ANOVA. *, p < 0.05

First, we wanted to determine if overexpression of N-Myc could sensitize a cell line to DON. We chose to use SK-N-AS cells for these experiments, which we have previously determined have no appreciable Myc expression (Fig. 2E). As shown by western blot analysis (Fig. 3A) SK-N-AS cells infected with pLOVE-NMyc lentivirus had elevated levels of NMyc protein compared to control cells. When these cells were treated with DON, we observed a significant shift to greater DON sensitivity in AS-NMyc cells compared to controls at the 1uM, 3uM, and 10uM concentrations of DON (Fig. 3B). These results suggest a direct causal relationship between NMyc expression levels and DON sensitivity.

**Figure 4:**
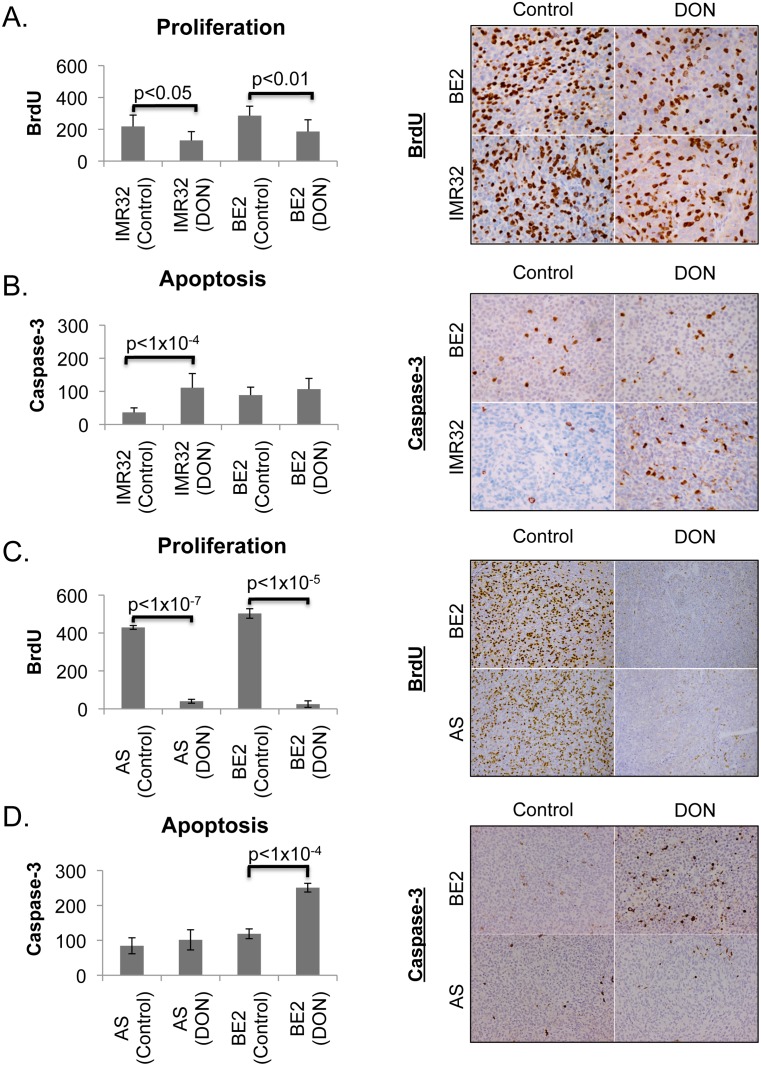
DON causes increased apoptosis in high-Myc, but not low-Myc expressing NBL tumors. (A and B) Immunohistochemistry (right panel) and quantification (left panel) of BrdU and cleaved Caspase-3 positive cells, respectively, in subcutaneous IMR32 and SK-N-BE2 tumors treated with 50mg/kg DON, twice weekly. (C and D) Immunohistochemistry (right) and quantification (left) of BrdU and cleaved Caspase-3 positive cells for both SK-N-AS and SK-N-BE2 tumors treated with 100mg/kg DON, twice weekly. Significance was determined by Student’s *t* test.

Prior studies suggest that interfering with glutamine utilization in nucleotide synthesis causes a Myc-independent loss in proliferation, while blocking glutaminolysis increases apoptosis in Myc overexpressing cell lines [8, 22, 23]. We therefore wanted to determine whether DON was affecting tumor growth by blocking proliferation or by increasing apoptosis. Tumors were isolated either when they had reached their maximal allowable size or when the mice reached their maximal allowable weight loss of 20%. Both BE2 tumors and IMR32 tumors were evaluated by IHC for markers of apoptosis (cleaved caspase-3) and proliferation (BrdU incorporation). DON was given 6-hrs prior and BrdU injected 2 hours prior to harvesting the tumors for histology. IMR32 tumors showed a ~50% reduction in BrdU incorporation (Fig. 4A) and an almost 3-fold increase in cleaved caspase-3 levels (Fig. 4B), while BE2 showed a 30% reduction in BrdU incorporation (Fig. 4A) and no significant changes in caspase 3 levels. These results indicate that DON at these doses had significant cytostatic and pro-apoptotic effects on IMR32 tumors, but only significant cytostatic effects on SK-N-BE2 tumors. Our results are consistent with prior studies showing that Myc expression levels are important for dictating the cellular response to inhibition of glutamine metabolism [7, 8, 24].

We next wanted to better determine the relationship between Myc expression levels and sensitivity to DON-induced apoptosis. Since BE2 is a N-Myc amplified neuroblastoma that has moderate N-Myc expression, we wanted to assess whether BE2 tumors lacked an apoptotic response to DON at the 50mg/kg dosage because of insufficient N-Myc expression or because of insufficient exposure to DON. We also wanted to confirm that tumors that do not overexpress Myc are resistant to DON-induced apoptosis. To study these related questions we set up additional NBL tumor studies using a higher dose of DON and the cell lines BE2 and AS, which we previously showed does not overexpress Myc (Fig. 2E). For these experiments we used a short course of treatment as we had already established that mice could not tolerate extended treatments at the 100mg/kg dose. Tumors in each cohort were allowed to grow to ~1000 mm^3^ before they underwent two treatments with 100mg/kg DON. Using the same timing as the previous tumor experiment, on day 4 when the second dose of DON was administered, DON was injected 6 hours and BrdU 2 hours prior to harvesting the tumors for histology. Our result showed that both BE2 and AS had a >90% reduction in BrdU incorporation after treatment with DON (Fig. 4C) showing a more profound cytostatic effect at this higher dose for BE2 then in the previous experiment. BE2 tumors now also showed a greater than 2-fold increase in caspase-3 cleavage, which was not previously seen at the lower dose, while AS tumors did not show an increase in caspase-3 activation (Fig. 4D). Our *in vivo* tumor studies indicate that with increasing Myc expression there is greater sensitivity to DON-induced apoptosis while cells that do not overexpress Myc are resistant to cell death from inhibition of glutamine metabolism.

### Caspase inhibitor QVD can partially reverse the effects of high DON concentration

**Figure 5:**
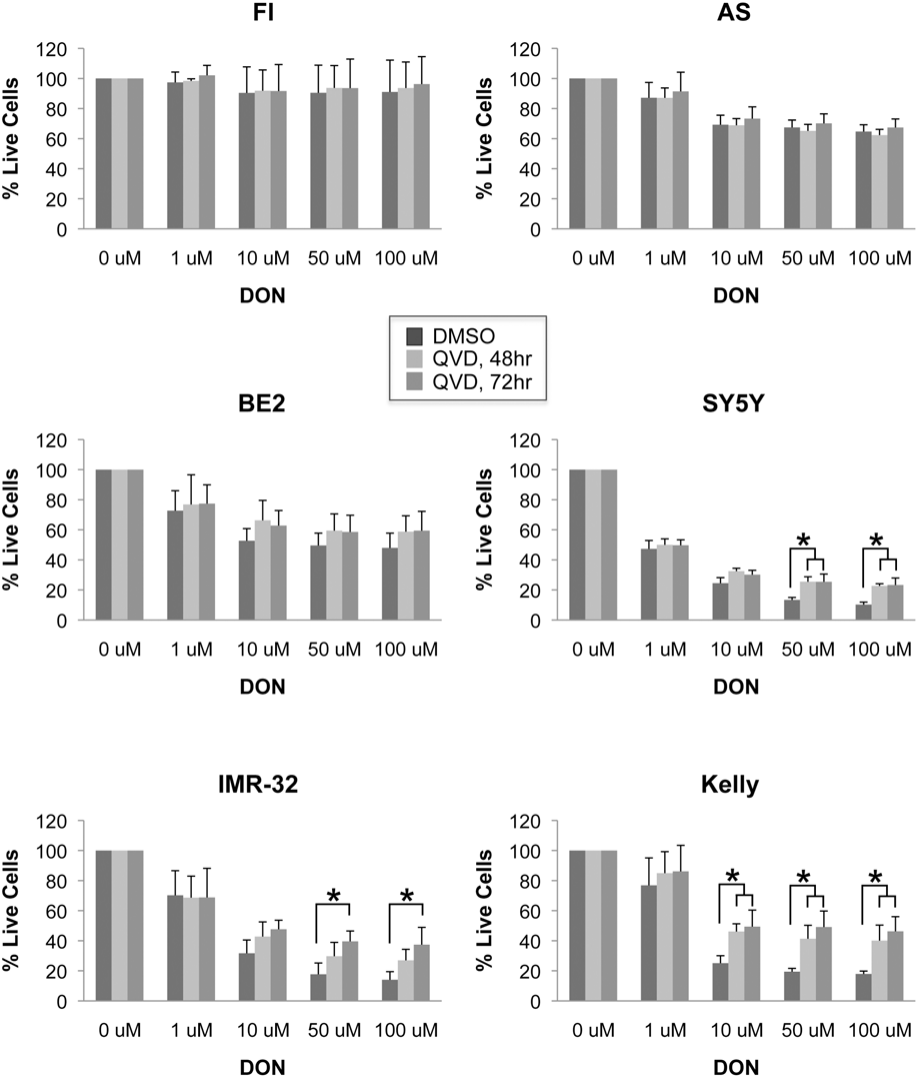
Inhibition of caspases partly reverses the effect of DON in high-Myc, but not low-Myc expressing cell lines. Cells were treated with DON for 72hrs and pan-caspase inhibitor QVD added 72hrs or 48 hrs prior to CyQuant assay for cell viability. Data is shown as average ± stdev, and is representative of 3–4 independent experiments. Statistical significance was determined by ANOVA. *, p < 0.05

To extend our observation that Myc expression sensitizes cells to DON-induced apoptosis we also tested whether the pan-caspase inhibitor QVD could partially interfere with DON’s effects on NBL cell lines *in vitro*. QVD was either added 48 hours before the end of a three-day assay or simultaneously with DON. Whether cells were exposed to 48 or 72 hrs of QVD, we observed significant rescue of cell viability after 10µM or higher DON treatment in the high N-Myc expressing cell lines IMR32 and Kelly, and the high c-Myc expressing cell line SY5Y (Fig. 5 and 2E). In contrast, QVD was not effective in rescuing cells treated with 1µM DON, suggesting that the observed decrease in cell number at this dose is caspase independent. Similar to our *in vivo* results, our *in vitro* QVD data show Myc expression sensitizes cells to DON-induced apoptosis. We did not observe a significant effect of QVD at the higher doses of DON in BE2 cells as might be expected from our *in vivo* results. This might reflect true differences between *in vivo* and *ex vivo* metabolism, or the effect might be masked from the higher amount of experimental error seen for that cell line.

### Knockdown of Bax prevents DON-induced apoptosis in Kelly cells

**Figure 6:**
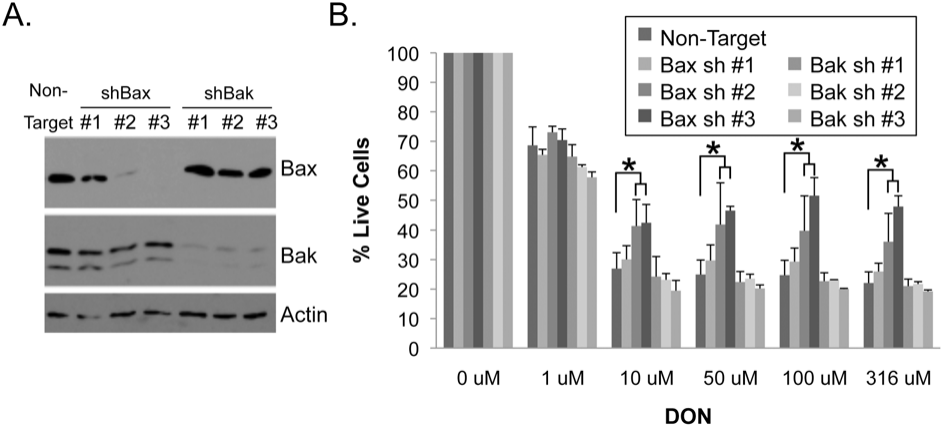
DON reduces cell viability through the pro-apototic Bax but not through Bak. (A) Kelly cells were infected with shRNA retrovirus to knockdown Bax and Bak. One week later, knockdown was assessed by western blot. (B) In parallel with western blot analysis, cells were treated with DON for an additional 72 hrs prior to measuring cell viability by CyQuant assay. Data is shown as average ± stdev, and is representative of 2 independent experiments. Significance was determined by ANOVA. *, p < 0.05

Though DON caused inhibition of tumor growth in all cell lines tested we want to augment apoptosis to promote tumor regression. Interference with glutamine metabolism is known to cause cellular stress that leads to apoptosis with a recent publication demonstrating that glutamine deprivation in neuroblastoma cell lines leads to cell death via Bax but not Bak [8]. To examine whether DON-induced apoptosis occurs through the same pathway, we used retrovirus to transduce Kelly cells with shRNA to knockdown Bax or Bak prior to DON treatment. As shown by western blot (Fig. 6A), Bax shRNA #2 and #3 led to substantial protein knockdown, and could partially reverse the effects of DON treatment (Fig. 6B). In contrast, significant knockdown of Bak with three individual shRNAs (#1-#3) was unable to rescue cells from DON (Fig. 6A and B). Similar to the QVD results for Kelly cells (Fig. 5) we see a reversal of DON effects only at the 10µM or higher concentration at which caspase-dependent death seems to occur. This confirms that Bax is an important mediator of cell death from loss of glutamine metabolism.

### Combination of DON with the Bcl-2 family antagonist ABT-263

**Figure 7:**
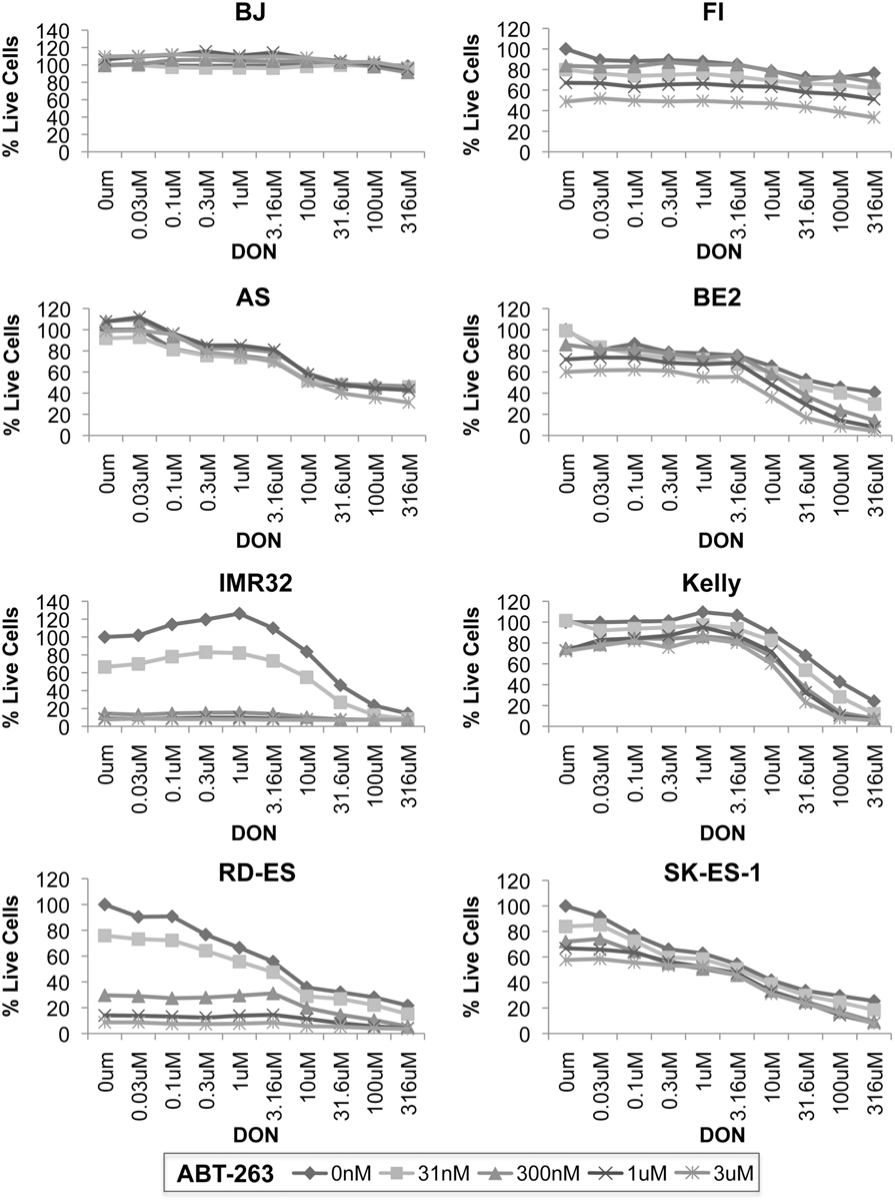
Combined treatment with DON and the Bcl-2 family antagonist ABT-263 is effective against NBL and EWS cell lines. Using a CyQuant assay, cell viability was determined following combined treatment with the indicated dose of DON and ABT-263 across a panel of NBL and Ewing’s sarcoma cell lines. BJ foreskin fibroblasts were used as a control. Percent live cells was determined by comparison to the no drug condition for each cell line. Data is representative of three independent experiments.

Though DON caused inhibition of tumor growth in all cell lines tested, induction of apoptosis was only observed in cells with high levels of Myc. Therefore, we were interested in identifying drugs that could cooperate with DON and enhance its ability to induce apoptosis and effect tumor regression. As the apoptotic effect of glutamine deprivation goes through the proapoptotic Bax protein and cancers often overexpress anti-apoptotic Bcl-2 family members, we decided to test DON in combination with the Bcl-2 family inhibitor ABT-263 (Navitoclax). ABT-263 is orally bioavailable, safe, and well tolerated, with thrombocytopenia as its major adverse side effect [25]. Combined treatment with DON and ABT-263 showed strong cooperative effects across a broad range of doses in virtually all of the cell lines tested (Fig. 7). We saw the greatest effect of DON and ABT-263 in cell lines with N-Myc expression (SK-N-BE2, IMR32 and Kelly) or c-Myc expression (RD-ES and SK-ES-1) (Fig. 2E and Fig. 7). Statistical analysis of DON and ABT-263 in the various cell lines was performed by the Department of Biostatistics at St. Jude Children’s Research Hospital with a statistical test to assess the differences in mean response between ABT-263 and DON on cell viability measured by CyQuant assay. This statistical method detects the deviation from the Loewe additive drug combination reference model [26]. By this method, synergistic effects were identified between ABT-263 and DON in SK-N-BE2 cells at 31μM or higher for DON and 300nM or higher for ABT-263. For Kelly cells, synergistic effects were identified at concentrations of 100µM or higher for DON and 1µM or higher for ABT-263, with all other doses showing additive effects. For all other cell lines, this drug combination showed additive effects. The promising outcomes we observed when DON was combined with ABT-263 indicate that DON-induced apoptosis depends on the balance of pro- and anti-apoptotic factors in the cell. These results suggest targeting glutamine metabolism while reducing the threshold for apoptosis is a very promising treatment strategy for neuroblastoma, Ewing’s sarcomas, and possibly other glutamine-addicted malignancies.

## Discussion

Oncogenic changes to cellular metabolism provide conditions which promote rapid proliferation. Recently, there has been renewed interest in understanding and exploiting these metabolic changes to develop novel anti-neoplastic therapies. In this study we interrogated the more common metabolic changes seen in cancers including acquired aerobic glycolysis, increased lactic acid production, increased fatty acid metabolism and increased glutamine metabolism. We were interested in identifying susceptible metabolic pathways in two pediatric cancers: neuroblastoma and Ewing’s sarcoma. We further wanted to determine whether xenograft tumors remained vulnerable to interference of those pathways *in vivo*, where differences in oxygen availability and nutrient resources might alter the metabolic needs of the cancer.

Aerobic glycolysis, the Warburg effect, is of central importance in cancer as it is almost a universal trait of the disease [3]. Consequently, many investigators have worked on identifying novel anti-metabolite based therapies that target enzymes in this pathway. Surprisingly, our initial screen showed NBL cell lines were not particularly sensitive to agents that target aerobic glycolysis (bromopyruvate, lonidamine, and sodium dichloroacetate) or lactic acid production (oxamate), which is necessary for maintaining high rates of glycolytic flux [16]. The only glycolytic inhibitor which showed some efficacy was bromopyruvate, which targets glyceraldehyde-3-phosphate dehydrogenase (GAPDH). However, this metabolic inhibitor was differentially toxic to control BJ cells and five of the cell lines tested at only one dose. Cancer cells also have high fatty acid demands for membrane production (fatty acid synthesis) and bioenergetics (fatty acid oxidation). Normal cells typically meet their needs from dietary sources, but many cancers show a dependency for either *de novo* fatty acid synthesis or increased fatty acid scavenging with augmented fatty acid oxidation [16, 27, 28]. Nevertheless, our attempts to target fatty acid oxidation (etomoxir and trimetazidine) or fatty acid synthesis (bezafibrate) were mostly ineffective, though etomoxir did show an effect at the highest dose tested. After glucose, glutamine is the next most catabolized nutrient in cancer cells, yet we are only just starting to understand its importance and regulation in cancer [4, 29]. Breakdown of glutamine not only provides a carbon source for the citric acid cycle, but also provides a source of nitrogen for a variety of anabolic processes such as nucleotide synthesis. Inhibiting glutamine metabolism may provide an effective way to simultaneously target multiple biosynthetic pathways. In contrast to the other metabolic pathways that we targeted, the inhibition of glutamine metabolism (6-diazo-5-oxo-L-norleucine) was effective across a broad panel of NBL and Ewing’s sarcoma cell lines, with all cells showing some DON sensitivity. *In vivo*, both EWS and NBL tumors were responsive to DON treatment with IMR32 showing almost a complete block in tumor growth. By using a focused screen of inhibitors to metabolic pathways commonly altered in cancer, we were able to demonstrate that both of these cancers are sensitive to inhibition of glutamine metabolism both *in vitro* and *in vivo*. In fact, SK-N-FI cells which were relatively unaffected by DON *in vitro*, were more responsive in tumor studies than we had anticipated suggesting that the *in vivo* environment may make tumors more sensitive to glutamine deprivation.

Oncometabolism is an emerging concept in the field of cancer that asserts oncogenic drivers dictate the specific metabolic strategies used by cancer cells in a particular tumor type [16]. The intertwining of oncogenes and metabolism suggests that oncogenic master regulators of metabolism could act as biomarkers for identifying patients who would likely benefit from a particular metabolic inhibitor. Recent studies have established that Myc oncogenes create a reliance on glutamine and have suggested interfering with glutamine utilization as a therapy for Myc-overexpressing cancers. By using a well-characterized inhibitor of glutamine metabolism (DON), we were able to confirm this observation *in vitro* showing Myc expression correlates with caspase-induced cell death caused by glutamine inhibition. Results from our *in vivo* studies further confirmed the correlation between Myc expression and DON-induced apoptosis: cells with high Myc expression were most sensitive to DON-induced apoptosis, cells with intermediate Myc expression needed higher concentrations of DON to elicit apoptosis, and two cell lines that do not overexpress Myc only had a cytostatic response. Additionally, we established that Ewing’s sarcomas are sensitive to glutamine inhibition *in vivo*. Our work and the work of others suggest that Myc expression could be used as a biomarker for segregating patients for treatment with glutamine antagonists. More importantly, we established that there is a sufficient therapeutic window between normal cells and cancer cells for glutamine inhibition to work *in vivo*.

In patients, the rate limiting side effect of DON in adults was weight loss as well as nausea. However, both these issues were mitigated using anti-emetics in pediatric patients. In our study, we saw that 50mg/kg of DON was an effective dose for controlling tumors in mice, while a short treatment with the less tolerated dose of 100mg/kg showed more profound cytostatic and pro-apoptotic effects but induced severe weight loss. In children a dose of 540mg/kg of DON was safely achieved without any rate-limiting toxicities, and this would be equivalent to 180mg/kg in the mouse [19]. This suggests that effective anti-glutamine therapies are possible even if nausea and weight loss are unavoidable side effects. Even more promising would be development of glutamine metabolism inhibitors that limit nausea. Our findings imply that under that circumstance a wider range of Myc expressing tumors would be susceptible to apoptosis from a higher treatment dose of glutamine inhibitors. This further supports the importance of finding novel glutamine metabolism inhibitors for targeted treatment of Myc-driven cancers.

The DON-induced activation of cell death via Bax led us to testing ABT-263, an antagonist to Bcl-2 family members which are direct inhibitors of Bax. Our experiments combining DON and ABT-263 showed promising additive and synergistic effects which suggest this could be an effective combination therapy *in vivo*. Though no Bcl-2 antagonists are currently FDA approved, there is a large class of these compounds moving through clinical trials increasing the likelihood of future FDA approval [30]. Furthermore, continued testing of other Bcl-2 antagonists should identify additional promising drugs capable of enhancing the effects of DON. Additionally, signaling through the PI3K/AKT survival pathway blocks proapoptotic signaling from Bax, therefore inhibitors of this pathway may also be good candidates to test in combination with inhibitors of glutamine metabolism [31]. Our results with DON support mounting evidence in the cancer metabolism field that targeting glutamine metabolism is a promising therapeutic strategy for Myc-overexpressing cancers, and identifying other inhibitors of glutamine metabolism could yield promising new therapies for pediatric patients. Since the failure of single agent therapies is a likely outcome of clinical studies the identification of a promising combinatorial therapy using glutamine antagonists and Bcl-2 antagonists provides an important path forward for future clinical studies.

## Supporting Information

S1 ARRIVE Checklist Animal Research: Reporting of In Vivo Experiments. (PDF)

S1 Fig The glutamine antagonist 6-diazo-5-oxo-L-norleucine is an effective inhibitor across a broad panel of NBL and EWS cell lines. Cell viability as a percent of control (% Live Cells) is graphed in a dose response curve following 72 hrs DON treatment across a panel of (A) NBL and (B) Ewing’s sarcoma cell lines using the immortalized BJ cell line as a control. Data shown are representative of three independent experiments.
(TIF)

S2 Fig DON significantly inhibits EWS xenograft tumor growth. (A) SK-N-MC and (B) SK-ES-1 tumors were treated with DON at 100 mg/kg or water by i.p. twice weekly. Weight loss in mice from DON reduced the treatment cohort to 2 mice indicated by (2) at later timepoints. Data is shown as percent relative tumor volume (% RTV), and statistical significance was determined by Student’s t-test.
(TIF)
