# Supplementary figures and images for: Antagonizing Bcl-2 Family Members Sensitizes Neuroblastoma and Ewing’s Sarcoma to an Inhibitor of Glutamine Metabolism
Source: PLoS One. 2015 Jan 23;10(1):e0116998. doi: 10.1371/journal.pone.0116998 (PMC4304844; doi:10.1371/journal.pone.0116998)

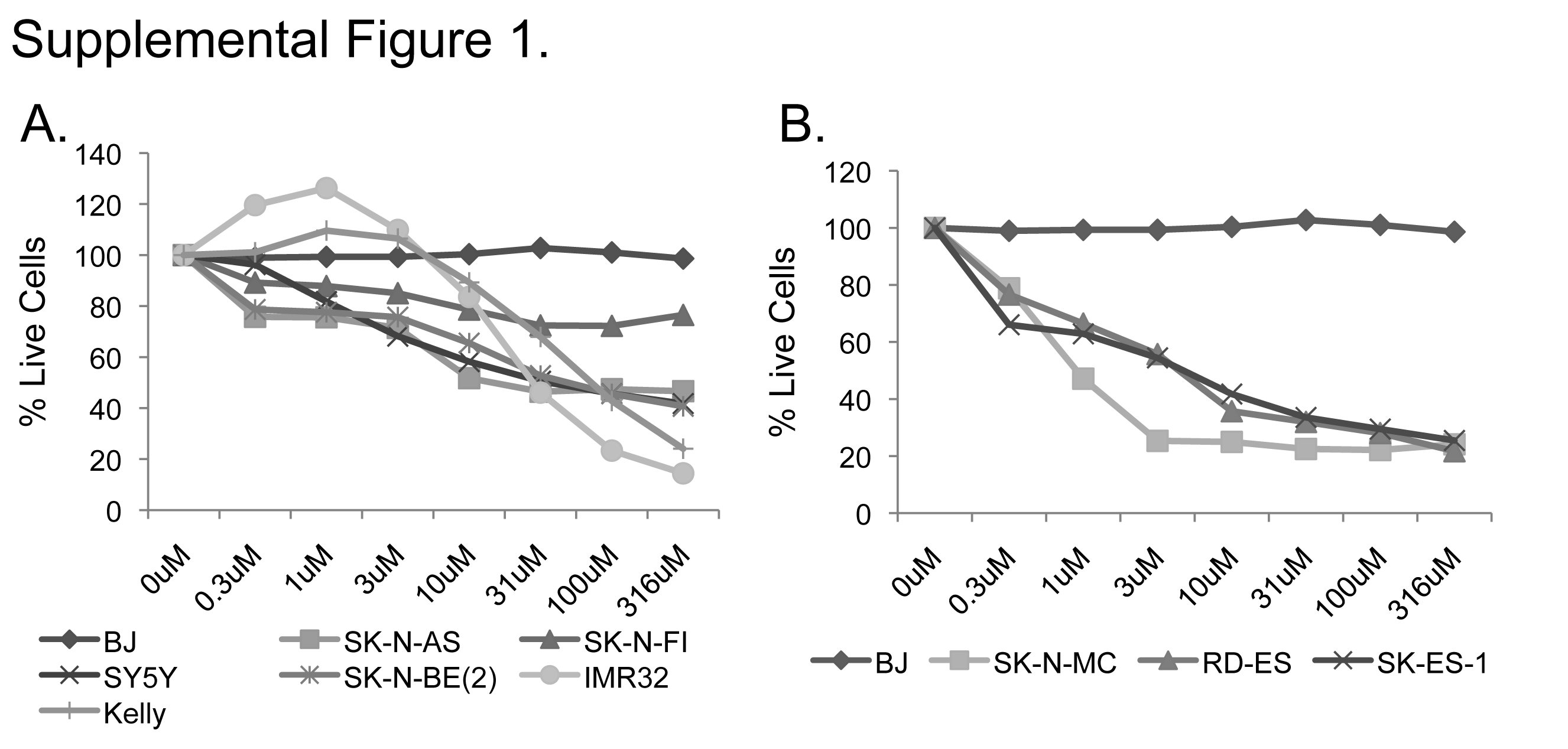

Supplement: S1 Fig — Cell viability as a percent of control (% Live Cells) is graphed in a dose response curve following 72 hrs DON treatment across a panel of (A) NBL and (B) Ewing’s sarcoma cell lines using the immortalized BJ cell line as a control. Data shown are representative of three independent experiments. (TIF) [file pone.0116998.s002.tif]

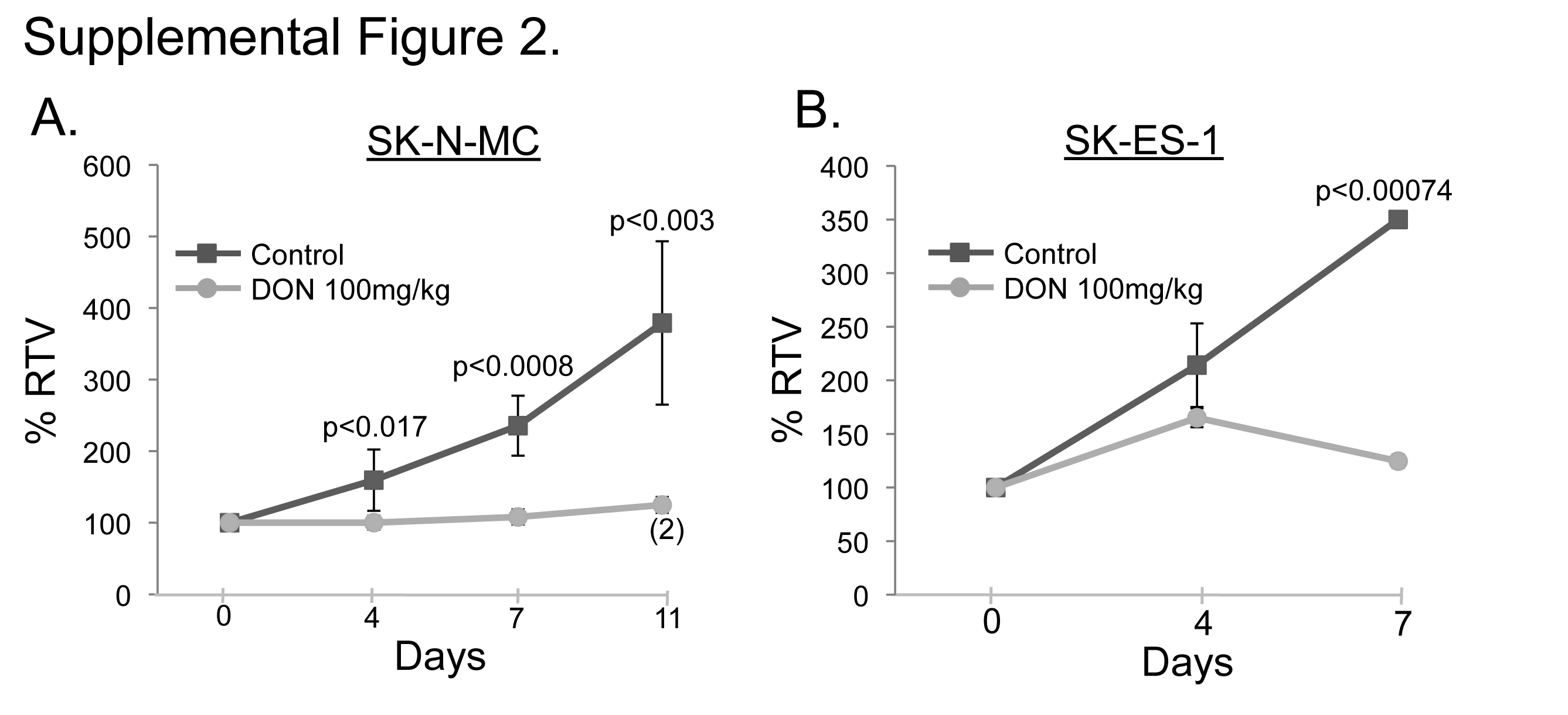

Supplement: S2 Fig — (A) SK-N-MC and (B) SK-ES-1 tumors were treated with DON at 100 mg/kg or water by i.p. twice weekly. Weight loss in mice from DON reduced the treatment cohort to 2 mice indicated by (2) at later timepoints. Data is shown as percent relative tumor volume (% RTV), and statistical significance was determined by Student’s t-test. (TIF) [file pone.0116998.s003.tif]
